# Heat Waves and Health Outcomes in Alabama (USA): The Importance of Heat Wave Definition

**DOI:** 10.1289/ehp.1307262

**Published:** 2013-11-22

**Authors:** Shia T. Kent, Leslie A. McClure, Benjamin F. Zaitchik, Tiffany T. Smith, Julia M. Gohlke

**Affiliations:** 1 Department of Environmental Health Sciences, and; 2 Department of Biostatistics, University of Alabama at Birmingham, Birmingham, Alabama, USA; 3 Department of Earth and Planetary Sciences, Johns Hopkins University, Baltimore, Maryland, USA

## Abstract

Background: A deeper understanding of how heat wave definition affects the relationship between heat exposure and health, especially as a function of rurality, will be useful in developing effective heat wave warning systems.

Objective: We compared the relationships between different heat wave index (HI) definitions and preterm birth (PTB) and nonaccidental death (NAD) across urban and rural areas.

Methods: We used a time-stratified case-crossover design to estimate associations of PTB and NAD with heat wave days (defined using 15 HIs) relative to non–heat wave control days in Alabama, USA (1990–2010). ZIP code–level HIs were derived using data from the North American Land Data Assimilation System. Associations with heat wave days defined using different HIs were compared by bootstrapping. We also examined interactions with rurality.

Results: Associations varied depending on the HI used to define heat wave days. Heat waves defined as having at least 2 consecutive days with mean daily temperatures above the 98th percentile were associated with 32.4% (95% CI: 3.7, 69.1%) higher PTB, and heat waves defined as at least 2 consecutive days with mean daily temperatures above the 90th percentile were associated with 3.7% (95% CI: 1.1, 6.3%) higher NAD. Results suggest that significant positive associations were more common when relative—compared with absolute—HIs were used to define exposure. Both positive and negative associations were found in each rurality stratum. However, all stratum-specific significant associations were positive, and NAD associations with heat waves were consistently positive in urban strata but not in middle or rural strata.

Conclusions: Based on our findings, we conclude that a relative mean-temperature-only heat wave definition may be the most effective metric for heat wave warning systems in Alabama.

Citation: Kent ST, McClure LA, Zaitchik BF, Smith TT, Gohlke JM. 2014. Heat waves and health outcomes in Alabama (USA): the importance of heat wave definition. Environ Health Perspect 122:151–158; http://dx.doi.org/10.1289/ehp.1307262

## Introduction

Climate change is expected to result in more frequent, more intense, and longer heat waves (Meehl and Tebaldi 2004). Increased mortality associated with heat waves is well documented (Basu 2009), and recent research suggests that heat waves may increase adverse birth outcomes (Basu et al. 2010; Strand et al. 2011a). However, most U.S.-based research examining associations between heat and health outcomes have focused on urban areas in the northeastern and midwestern regions. Furthermore, there has been little published on whether national heat wave warning systems are appropriate for all regions. The southeastern United States, particularly in the Deep South States of Alabama (AL), Mississippi, Louisiana, South Carolina, and Georgia, compared with the rest of the United States, have different climate patterns characterized by long, hot summers with higher minimum temperatures and humidity. There are also profound differences in demographic composition, quality and characteristics of housing, and urban–rural land-use and vegetation patterns (Bonan 1997; Brown et al. 2005; Golant and LaGreca 1994).

Previous studies that examined associations between heat waves and health outcomes have used a variety of heat wave metrics, making it difficult to compare results or determine the most appropriate metric for public health warning systems. Heat wave indices (HIs) are defined using temperatures alone or temperatures plus other meteorological factors, such as humidity and wind speed, and have either absolute or relative temperature thresholds that must be exceeded for a specified duration, ranging from one to several consecutive days (Smith et al. 2013). In an examination of 16 previously published HIs for the period 1979–2011 in the United States, Smith et al. (2013) observed substantial differences in heat wave geographic patterns and time trends by HI definition. Previous studies have reported stronger positive associations between heat waves and mortality in northeastern and midwestern cities compared with southeastern cities (Anderson and Bell 2011; Basu 2009), which suggests that acclimatization may contribute to region-specific exposure–response relationships, and that heat wave metrics and warnings may need to be regionally specified (Barnett et al. 2010; Bobb et al. 2011; Vaneckova et al. 2011).

Several studies have highlighted the need to increase our understanding of extreme heat events and adaptation strategies in rural versus urban areas (Huang et al. 2011; Knowlton et al. 2007; Reid et al. 2009). Evidence from previous studies has suggested that excess mortality and morbidity due to extreme heat events in urban environments are related to vulnerable populations and the urban heat island effect (Basu 2009; Huang et al. 2011; Kovats and Hajat 2008). Rural communities, although not as well studied as urban communities, may have unique vulnerabilities, such as increased distance to care, greater time spent outdoors, and lack of heat wave response systems (e.g., cooling centers) (Merwin et al. 2006; Weisskopf et al. 2002). Land-use patterns and demographic compositions in Deep South urban and rural regions differ from regions analyzed in previous heat wave health studies. In particular, the Deep South states have more poorly maintained housing stock, higher poverty, and both urban and rural areas with large non-Hispanic African–American populations (Golant and LaGreca 1994).

We analyzed Alabama birth and death records to examine *a*) whether the use of different HIs results in differing associations between heat waves and health outcomes, and *b*) whether associations between heat waves and health outcomes differ by rurality.

## Methods

*Vital records and outcomes*. We obtained records of live births and deaths for May–September of 1990–2010 from the Alabama Department of Public Health (ADPH; Montgomery, AL). The study protocol was reviewed and approved by the ADPH Institutional Review Board and the University of Alabama at Birmingham Institutional Review Board (protocol #X110706005).

Live birth records included the date of birth, gestational age, birth weight, and the mother’s residential ZIP code. After excluding 81 births with unlikely weights (< 200 g) (Wolf and Armstrong 2012), 62,803 (of 543,980 births) were classified as preterm births (PTBs), having gestational ages between 24 and 37 weeks (Morgan et al. 2008). Of these, 60,466 PTBs had birthdates, residences in Alabama ZIP codes, and available meteorological and rurality data.

Death records indicated the date of death, the deceased’s residential ZIP code, and the cause of death coded by the *International Classification of Diseases* 9th (ICD-9) or 10th (ICD-10) revision (World Health Organization 1977, 2010). Of 381,776 deaths in Alabama (1990–2010), 347,432 deaths were nonaccidental (ICD-9 codes < 800, and ICD-10 codes with letters A–R) (Crouse et al. 2012). Of these, 301,126 nonaccidental deaths (NADs) had associated dates, residences in Alabama ZIP codes, and available meteorological and rurality data.

*HIs*. Sixteen HIs were identified from the public health and climate change literature (Anderson and Bell 2009; Anderson and Bell 2011; Hattis et al. 2012; Meehl and Tebaldi 2004; Peng et al. 2011; Robinson 2001; Rothfusz 1990; Steadman 1979, 1984; Tan et al. 2007), as well as from indices used by the National Weather Service to develop a public warning system (Rothfusz 1990; Steadman 1979). Complete definitions, calculation methods, and heat wave geographical patterns of these 16 HIs by six continental U.S. regions and temporal trends during 1979 to 2011 have been described previously (Smith et al. 2013), and short definitions are shown in Table 1. Briefly, meteorological data (temperature, specific humidity, surface pressure, surface downward shortwave radiation, surface downward longwave radiation, and directional wind components) on a 12.5-km grid for 1990–2010 were obtained by Smith et al. (2013) from Phase 2 of the North American Land Data Assimilation System (NLDAS-2) (Cosgrove et al. 2003) and used to develop daily ZIP code–level HI estimates for the current study. Heat wave days were identified at the ZIP code level using each day’s meteorological variables. Relative HIs were based on the individual ZIP code’s 1990–2010 meteorological history. If > 50% of land area within a ZIP code was above the specific HI threshold on a given day, the ZIP code was determined to be in a heat wave. ZIP codes, obtained from either the mother’s residence (birth records) or deceased’s residence (death records), were used in combination with the event day (i.e., the day of preterm birth or the day of death) to merge vital records with the census ZIP code tabulation area–based HIs. HI16 was eliminated from further analysis because no heat wave days occurred in Alabama during the study period according to this definition. For each of the 640 ZIP codes used, the mean area (± SD) was 226 km^2^ ± 200.

**Table 1:** Summary of data on HIs, PTB (*n* = 60,466), and NAD (*n* = 301,126) in 640 Alabama ZIP codes during 1990–2010.

HI	Definition	Reference	HI days/year/ZIP [*n* (%)]^*a*^	PTB [*n* (%)]	NAD [*n* (%)]
HI01	Mean daily temperature > 95th percentile for ≥ 2 consecutive days	Anderson and Bell 2011	1.34 (0.9)	652 (1.1)	2,678 (0.9)
HI02	Mean daily temperature > 90th percentile for ≥ 2 consecutive days	Anderson and Bell 2011	5.41 (3.5)	2,373 (3.9)	10,463 (3.5)
HI03	Mean daily temperature > 98th percentile for ≥ 2 consecutive days	Anderson and Bell 2011	0.18 (0.2)	111 (0.2)	444 (0.2)
HI04	Mean daily temperature > 99th percentile for ≥ 2 consecutive days	Anderson and Bell 2011	0.01 (0.0)	1 (0.0)	11 (0.0)
HI05	Minimum daily temperature > 95th percentile for ≥ 2 consecutive days	Anderson and Bell 2011	0.08 (0.1)	44 (0.1)	104 (0.0)
HI06	Maximum daily temperature > 95th percentile for ≥ 2 consecutive days	Anderson and Bell 2011	3.54 (2.3)	1,610 (2.7)	7,385 (2.5)
HI07	Maximum daily temperature ≥ 81st percentile every day, ≥ 97.5th percentile for ≥ 3 nonconsecutive days, and consecutive day average ≥ 97.5th percentile	Peng et al. 2011	1.77 (1.2)	839 (1.4)	4,106 (1.4)
HI08	Maximum daily apparent temperature^*b*^ > 85th percentile for ≥ 1 day	Hattis et al. 2012; Steadman 1984	19.33 (12.6)	8,333 (13.8)	37,169 (12.3)
HI09	Maximum daily apparent temperature^*b*^ > 90th percentile for ≥ 1 day	Hattis et al. 2012; Steadman 1984	10.91 (7.1)	4,681 (7.7)	21,018 (7.0)
HI10	Maximum daily apparent temperature^*b*^ > 95th percentile for ≥ 1 day	Hattis et al. 2012; Steadman 1984	3.51 (2.3)	1,568 (2.6)	6,826 (2.3)
HI11	Maximum daily temperature > 35°C (95°F) for ≥ 1 day	Tan et al. 2007	1.43 (0.9)	497 (0.8)	2,276 (0.8)
HI12	Minimum daily temperature > 26.7°C (80.1°F) or maximum daily temperature > 40.6°C (105.1°F) for ≥ 2 consecutive days	Robinson 2001	2.90 (1.9)	1,203 (2.0)	5,701 (1.9)
HI13	Maximum daily heat index^*c*^ > 80°F for ≥ 1 day	Rothfusz 1990; Steadman 1979	125.47 (82.1)	50,176 (83.0)	245,833 (81.6)
HI14	Maximum daily heat index^*c*^ > 90°F for ≥ 1 day	Rothfusz 1990; Steadman 1979	78.26 (51.2)	31,495 (52.1)	151,189 (50.2)
HI15	Maximum daily heat index^*c*^ > 105°F for ≥ 1 day	Rothfusz 1990; Steadman 1979	3.35 (2.2)	1,368 (2.3)	5,581 (1.9)
HI16	Maximum daily heat index^*c*^ > 130°F for ≥ 1 day	Rothfusz 1990; Steadman 1979	NA	NA	NA
NA, not applicable. ^***a***^Percentages of HI days/year/ZIP code were calculated using the 153 days in May–September as the denominator. ^***b***^Apparent temperature is a function of air tempera­ture, humidity, wind speed, and solar radiation. ^***c***^The HI is a function of air temperature and humidity, parameterized to take account of other environmental factors.					

*Rurality measures*. Vital records were merged with two ZIP code–level measures of rurality. First, Rural-Urban Commuting Area Codes (RUCA), version 2.0 (Hart et al. 2005) were classified using the suggested “categorization B,” which divides among “urban focused,” “large rural city/town (micropolitan) focused,” and “small rural and isolated town focused” categories. The rural RUCA category, consisting of areas with low levels of commuters who travel to places with populations ≥ 10,000, represents populations most economically disconnected from cities and larger towns (Hart et al. 2005; Kent et al. 2013). Second, Census 2000 population densities (U.S. Census Bureau 2000) were classified into tertiles, with the highest tertile capturing the most population-dense urban areas. In addition to these measures of rurality, summertime maximum 16-day green vegetation fraction (GVF), a measure of percent live vegetation cover, was derived from Moderate Imaging Resolution Spectrometer (MODIS) satellite sensor data at 1-km resolution (Zeng et al. 2000). To calculate a ZIP code–level GVF value, we took an average of 1-km GVF grid cells that had centroids within a ZIP code. GVF was calculated for 2004, a typical, nondrought year in Alabama; GVF was divided into tertiles for analyses.

*Study design and analysis*. We used a time-stratified case-crossover design (Basu et al. 2005; Crouse et al. 2012; Janes et al. 2005b). A case-crossover design emulates a retrospective nonrandomized crossover study; in this design each case event (either PTB or NAD) is matched with a counterfactual control exposure period (Maclure 1991). In the time-stratified sampling design, all days that are on the same day of the week and within the same month as the case day are selected as control periods. The time-stratified control selection method is frequently used in environmental health studies because it controls for time trends, seasonality, and overlap bias (Basu et al. 2005; Crouse et al. 2012; Janes et al. 2005a, 2005b; Tong et al. 2012). Case-crossover data are analyzed in the same manner as a matched case–control design, using the case and matched time-stratified control periods as stratum in conditional logistic regression models (Wang et al. 2011). Analyses were run using SAS 9.3 (SAS Institute Inc., Cary, NC), and splines in conditional logistic regression models were performed using the LGTPHCURV9 macro (Li et al. 2011).

*Lags, duration, and seasonality*. To determine whether associations of PTB and NAD with heat waves varied according to characteristics of the heat wave, we examined associations of each outcome with heat wave days (defined using the 15 HIs) versus non–heat wave control days on the same day as the event (i.e., the day of birth or death, lag0) and up to 6 days before the event (lag1–lag6). Previous studies have reported associations between acute morbidity and heat within a few days of the outcome, with days further back in time carrying null associations (Ye et al. 2012). To compare our results with those of previous studies, we examined whether lag0 associations changed when we adjusted for daily mean temperature as a natural cubic spline with three degrees of freedom and equally spaced knots (Anderson and Bell 2009). We performed separate analyses to determine whether the timing of the heat wave influenced associations. Specifically, we ran models that included a product term between heat wave days and a variable indicating whether the heat wave day occurred during the first heat wave of the season; we also ran a second set of models that included product terms between heat wave days and a variable indicating whether the heat wave occurred early in the season (May–July) or late in the season (August–September). To examine modification of associations according to the duration of heat waves, we determined the median length of heat waves (in each of our birth record and death record samples) defined according to each HI, and estimated associations separately for heat wave days during heat waves that were shorter than the median duration and for heat wave days during heat waves that were longer than the median duration. We used conditional logistic regression models to estimate odds ratios (ORs) and 95% confidence intervals (CIs) for the associations between heat wave days and PTB or NAD; associations are reported as the percent difference in the odds of the outcome on heat wave days compared with non–heat wave days [percent difference = (OR – 1) × 100]. We used Akaike information criterion (AIC) values to estimate relative goodness of fit across the models (Bozdogan 1987).

*Estimating model parameter differences*. Based on AIC values and previous applications of the HIs, we selected six HIs to examine further. To estimate whether there were significant differences in parameter estimates across the models, we calculated bootstrapped bias-corrected percentile-based CIs. We calculated the CIs using 999 samples with replacement and determined the bias-corrected 2.5 and 97.5 percentiles (Ajmani 2009; Carpenter and Bithell 2000; UCLA: Statistical Consulting Group 2012). If a bootstrapped 95% CI for the difference between two point estimates on the lnOR scale did not include zero, we considered the two estimates to be significantly different (*p* < 0.05).

*Estimating effect modification by rurality*. We modeled multiplicative interaction terms between RUCA categories and heat wave days to determine whether associations differed among ZIP codes classified as urban, large town, or small town. In addition, we estimated associations according to tertiles of population density and GVF as alternative measures of rurality.

## Results

*Distribution of heat waves and cases on heat wave days across HIs*. Table 1 shows that the numbers of PTBs and NADs occurring during heat waves varied depending on the HI definition used. For example, when heat waves were defined using HI04 (mean daily temperature > 99th percentile for ≥ 2 consecutive days), each ZIP code had an average of 0.01 heat wave days/season, resulting in a total of 1 PTB and 11 NADs on heat wave days during 1990–2010. In contrast, when heat waves were defined using HI13 (maximum daily heat index > 80°F for ≥ 1 day), each ZIP code had an average of approximately 125 heat wave days/season (82% of the 153 days from May to September), and > 80% of all PTB and NAD occurred on days classified as a heat wave day.

**Figure 1:**
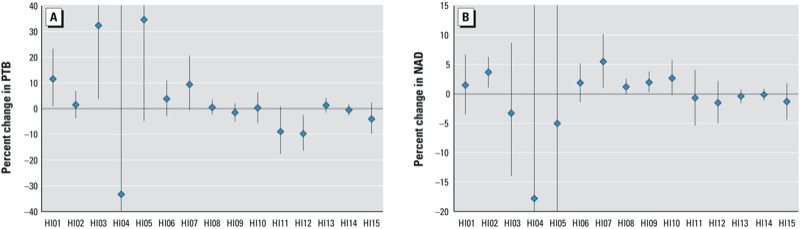
Percent change (95% CI) in PTB (*A*) or NAD (*B*) on the day of a heat wave (lag0) compared with corresponding non–heat wave control days, by 15 HIs. Estimates are derived from ORs and 95% CIs estimated using unadjusted case-crossover conditional logistic regression models. See Supplemental Material, Table S2, for corresponding numeric data.

**Figure 2:**
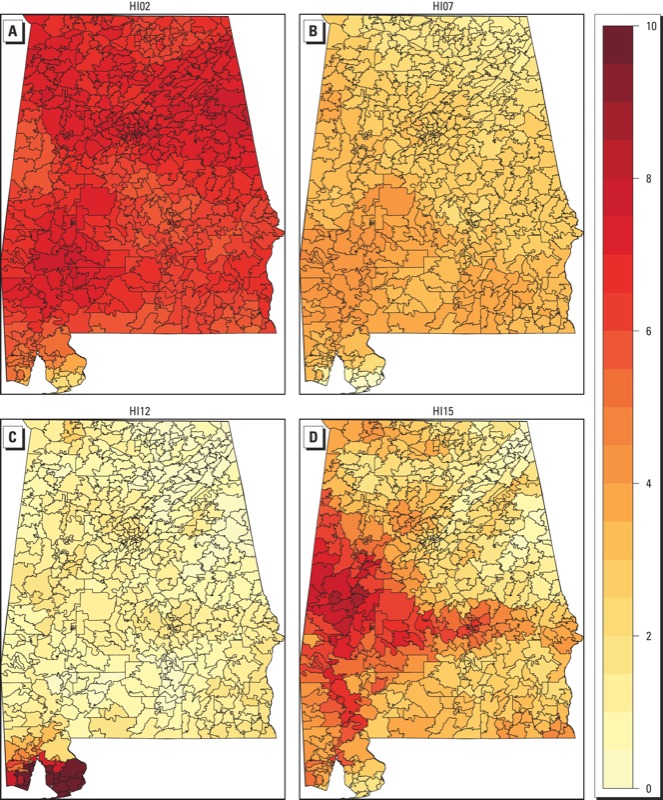
Number of Alabama ZIP code–level average HI days per year (indicated by the color scale) by (*A*) HI02, (*B*) HI07, (*C*) HI12, and (*D*) HI15. Heat waves are defined as follows: HI02, mean daily temperatures > 90th percentile for ≥ 2 consecutive days; HI07, maximum daily temperatures ≥ 81st percentile every day, ≥ 97.5th percentile for ≥ 3 non­consecutive days, and consecutive day average ≥ 97.5th percentile; HI12, minimum daily temperatures > 26.7°C (80.1°F) or maximum daily temperature > 40.6°C (105.1°F) for ≥ 2 consecutive days; and HI15, maximum daily heat index values > 105°F for ≥ 1 day. The Gulf of Mexico borders the southwestern corner of Alabama, with the Florida panhandle separating the ocean on the south­eastern corner. Western, northern, and eastern Alabama borders other states.

*Associations with heat waves defined using different HIs*. Associations between heat wave days and PTB were positive for heat waves defined by 9 of the 15 HIs when models were not adjusted for mean daily temperature (Figure 1A; see also Supplemental Material, Table S1). Positive associations with heat waves defined using HI01 (mean daily temperature > 95th percentile for ≥ 2 consecutive days) and HI03 (mean daily temperature > 98th percentile for ≥ 2 consecutive days) were statistically significant, with 11.6% (95% CI: 0.9, 23.4%) and 32.4% (95% CI: 3.7, 69.1%) higher odds of PTB on heat wave versus non–heat wave days, respectively. Associations with heat wave days defined using HIs based on mean daily temperatures increased as the threshold temperature increased from the 90th percentile (HI02, 1.5% higher PTB; 95% CI: –3.5, 6.9%) to the 95th and 98th percentiles (HI01 and HI03, respectively), but PTB was negatively associated with heat wave days when the mean daily temperature threshold was > 99th percentile (HI04, 33.3% lower PTB; 95% CI: –92.4, 453.9). However, as noted above, using the more stringent HI04 definition, only one PTB occurred during the study period. When heat waves were defined using HI12, which requires exceeding both minimum and maximum daily temperature thresholds for ≥ 2 consecutive days, the odds of PTB was significantly lower on heat wave days compared with non–heat wave days (9.7% decrease; 95% CI: –16.4, –2.5%). Although a total of 1,203 PTBs occurred on heat wave days, defined using HI12, the heat waves were almost entirely limited to ZIP codes in the southwest corner of Alabama, on the Gulf of Mexico (Figure 2C).

Associations between heat wave days and NAD were positive for heat waves defined by 7 of the 15 HIs (without adjustment for mean temperature) (Figure 1B; see also Supplemental Material, Table S1). Associations were statistically significant for heat waves defined by 3 of the HIs (HI02, HI07, and HI09). Specifically, the odds of NAD were higher in association with heat wave days defined by HI02 (mean daily temperature > 90th percentile for ≥ 2 consecutive days, 3.7% higher; 95% CI: 1.1, 6.3%), HI07 (based on 2 maximum daily temperature percentile cutoffs for at least 3 consecutive days, 5.5% higher; 95% CI: 1.0, 10.2%), and HI09 (maximum daily apparent temperature > 90th percentile for ≥ 1 day, 2.0% higher; 95% CI: 0.3, 3.8%). Associations between NAD and heat wave days defined using HIs based on maximum daily apparent temperatures increased as the threshold apparent temperature increased from the 85th percentile (HI08, 1.2% higher NAD; 95% CI: –0.1, 2.5%) to the 90th (HI09, 2.0% higher NAD; 95% CI: 0.3, 3.8%) and 95th percentiles (HI10, 2.7% higher NAD; 95% CI: –0.2, 5.7%). We found no evidence of a positive exposure response for PTB and apparent temperature HIs.

When models were adjusted for mean daily temperature, we observed no statistically significant positive associations between heat wave days and PTB or NAD (for any HI), and adjustment moved some positive associations closer to the null (i.e., HI01 and HI03 heat wave days with PTB; and HI02, HI07, and HI09 heat wave days with NAD) (see Supplemental Material, Table S1). Others changed from positive to negative, and the negative association with heat wave days defined using HI09 became statistically significant (4.7% lower odds of PTB, 95% CI: –8.5, –0.6%). However, adjusting for mean daily temperature had little effect on associations between heat wave days and PTB or NAD when heat waves were defined based on absolute values [vs. relative (percentile) thresholds] for heat index (HI13, HI14, HI15), maximum daily temperature (HI11), or minimum and maximum daily temperatures (HI12).

**Figure 3:**
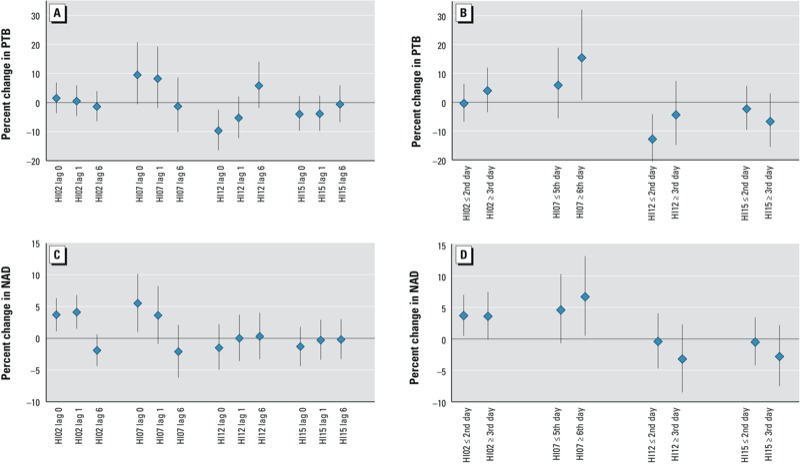
Percent change in PTB (*A,B*) or NAD (*C,D*) risks by selected HI lag day (*A,C*) and heat wave duration (*B,D*) using HI02, HI07, HI12, and HI15. Estimates are derived from ORs and 95% CIs estimated using unadjusted case-crossover conditional logistic regression models. Heat wave length cut points were determined using the median heat wave length for each HI. See Supplemental Material, Table S1, for corresponding numeric data.

*Differences in associations according to heat wave qualities*. We next evaluated modification of associations according to lags, heat wave duration, and seasonality (without adjustment for mean temperature). Of 10 relative HI lag0 models, 2 had significant relationships with PTB and 3 had significant relationships with NAD (Figure 1; see also Supplemental Material Table S1). All significant relative HI associations were positive. Of 5 absolute HI lag0 models, 1 had a significant negative relationship with PTB and none had significant relationships with NAD. Associations did not change from positive to negative with longer lags, as would be expected with mortality displacement (Figure 3; see also Supplemental Material, Figure S1). Longer duration heat waves numerically increased point estimates of associations (i.e., closer to the null for negative associations and further from the null for positive associations) with health outcomes for 10 of 15 HIs examined in PTB duration models and 7 of 15 HIs examined in NAD duration models (see Supplemental Material, Figure S2). Associations between heat wave days and the outcomes were generally consistent between the first heat wave of the season and subsequent heat waves, or between early- and late-season heat waves (data not shown).

*Evaluation of model fits and differences in parameter estimation across HIs.* The AIC is a relative measure of information lost when fitting the model; hence, smaller AIC values suggest better fitting models. AIC values differed by > 2 for models containing different HIs (see Supplemental Material, Table S2), indicating that there are likely differences in model fits (Burnham and Anderson 2004). Models of heat waves defined using HI02 had the lowest AIC across the NAD models, whereas models of heat waves defined using HI12 carried the lowest AIC among all PTB models. Across PTB models, models of heat waves defined using HI01 and HI03 had similar fits (AIC differences of 2.35 and 1.92, respectively) to models including HI12. HI12 had a negative association with PTB, whereas HI01 and HI03 had positive associations with PTB. Across NAD models, models of heat waves defined using HI07 and HI09 had similar fits (AIC differences of 2.03 and 2.55, respectively) to models including HI02. HI02, HI07, and HI09 all had positive associations with NAD.

HIs 02, 03, 07, 09, 12, and 15, which offer a representation of the different types of HIs used to define heat waves in previous epidemiological analyses and for heat wave warning systems, were examined in further detail. Bias-corrected percentile-based bootstrapped CIs indicate that in PTB models using HI03, associations were significantly numerically higher than those using HI09, HI12, or HI15 associations, whereas HI12 associations were significantly numerically lower than estimates for HI02 and HI07. For NAD models, HI07 associations were significantly numerically higher than those for HI12 and HI15.

*Differences in associations according to rurality categories*. Because the HIs based on mean daily temperature (HI01–HI03) had simple definitions at different relative temperature cut points for defining a heat wave and also had significant associations with adverse outcomes (HI01 and HI03 for PTB, HI02 for NAD), we chose these heat wave metrics to examine whether associations differed by rurality.

**Figure 4:**
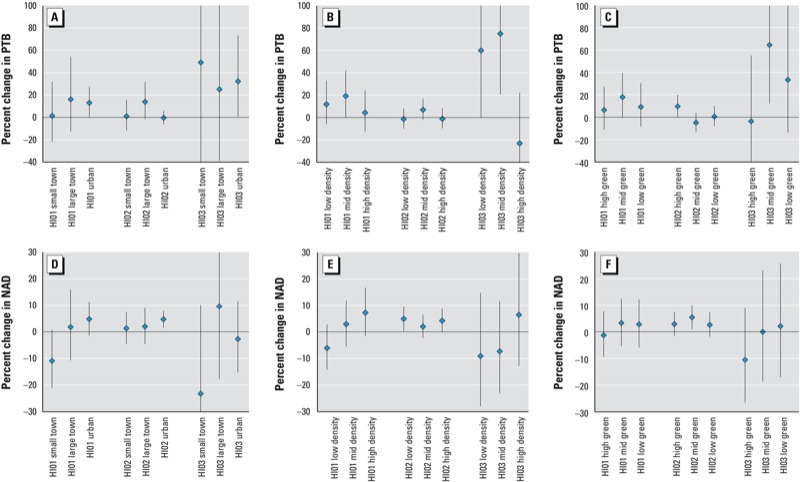
Percent increase in PTB (*A–C*) or NAD (*D–F*) on a heat wave day by HI (HI01, HI02, or HI03) stratified by (*A,D*) RUCAs, (*B,E*) population density tertiles, and (*C,F*) green vegetation factor tertiles. Estimates are derived from ORs and 95% CIs estimated using unadjusted case-crossover conditional logistic regression models.

All statistically significant rurality-specific associations between heat wave days and PTB were positive (Figure 4A–C). Heat wave days defined by HI03 were positively associated with PTB in ZIP code areas with populations in the lowest (59.9% increase; 95% CI: –0.5, 155.6%) and middle (75.0% increase; 95% CI: 20.7, 153.8%) tertiles of population density, but were negatively associated with PTB in ZIP code areas with populations in the highest tertile of population density (23.1% decrease; 95% CI: –51.6, 22.2%) (for HI03 and population density, *p* = 0.02) (Figure 4B). Heat wave days defined by HI02 were positively associated with PTB in ZIP code areas with the highest vegetation (9.6% increase; 95% CI: 0.1, 20.1%), but there was little evidence of associations for ZIP codes with medium (4.9% decrease; 95% CI: –13.0, 4.0%) or low vegetation (0.5% increase; 95% CI: –8.3, 10.1%) (for HI02 and GVF, *p* = 0.09) (Figure 4C). All other HI*rurality interaction terms in PTB models had *p*-values > 0.10.

All statistically significant rurality-specific associations between heat wave days and NAD were positive (Figure 4D–F). Associations between heat waves and NAD were consistently positive in the urban category for every HI*rurality model, except for the association between HI03 heat wave days and NAD in the RUCA urban category (2.7% decrease; 95% CI: –15.2, 11.6%), but the interaction term in this model was not significant (for HI03 and RUCA interaction, *p* = 0.32) (Figure 4D). Urban categories were consistently numerically higher in heat-wave-by-rurality models with interaction *p*-values ≤ 0.10. HI01 heat wave days showed a positive association with NAD in RUCA urban regions (4.8% increase; 95% CI: –10.9, 10.9%), an association closer to the null in RUCA large town regions (1.8% increase; 95% CI: –10.7, 16.0%) and a negative association in RUCA small town regions (10.9% decrease; 95% CI: –21.1, 0.7%) (for HI01 and RUCA interaction, *p* = 0.07) (Figure 4D). HI01 heat wave days showed a positive association with NAD in the most population-dense tertile (7.2% increase; 95% CI: –1.5, 16.7%) and the mid-density tertile (2.9% increase; 95% CI: –5.5, 11.9%), but a negative association in the least population-dense tertile (76.1% decrease; 95% CI: –14.2, 2.8%) (for HI01 and population density interaction, *p* = 0.10) (Figure 4E). All other heat wave by rurality interaction terms in NAD models had *p*-values > 0.10.

## Discussion

In the present study, we examined which of 16 HIs were most predictive of two important adverse health outcomes, PTB and NAD, using novel exposure and health outcome data sets covering both urban and rural areas in Alabama. Compared with non–heat wave control days, heat wave days defined using HI02 were associated with 3.7% (95% CI: 1.1, 6.3%) higher NAD, and heat wave days defined using HI03 were associated with 32.4% (95% CI: 3.7, 69.1) higher PTB. After adjusting for temperature, these positive associations were attenuated, suggesting that increases in temperature can account for some of the effects observed during heat waves.

*PTB results compared with previous findings*. Relationships between birth outcomes and heat waves have not been studied extensively. In a study in California that also used a case-crossover design, Basu et al. (2010) reported that a 10°F increase in weekly temperatures was associated with an 8.6% increase in PTB. Other studies have reported a null association between temperature and gestation length and indicated that previous associations might be attributed to uncorrected bias in cohort study analyses (Strand et al. 2011a, 2011b; Wolf and Armstrong 2012). The present study suggests that there may be a positive association between PTB and heat wave days defined using a percentile-based mean daily temperature metric (HI01 and HI03). Associations between PTB and heat wave days defined using other metrics were not significant, with the exception of a significant negative association with heat wave days based on a previously used metric (HI12), which in Alabama exhibited heat waves only in a small region of the state located on the Gulf of Mexico (Figure 2C). Region might play a role in differences in associations: Basu et al. (2010) found differences in associations between heat waves and PTB across 16 counties in California.

*Comparison of NAD results with previous findings*. Using data from 43 cities, Anderson and Bell (2011) found an average 3.7% increase in mortality associated with a heat wave defined as ≥ 2 consecutive days with mean temperatures > 95th percentile during May through September (HI01); however, when cities were separated by region, the authors found a lower, nonsignificant association in the Southeast (1.8% increase; 95% CI: –0.11, 3.84%). Our unadjusted results showed a similar association. However, after adjusting for daily mean temperature, the point estimate fell below zero (–2.2%; 95% CI: –7.4, 3.3%). Peng et al. (2011) examined the association between heat wave days, defined using HI07, and mortality in Chicago, Illinois, and reported a temperature-adjusted 7.8% higher mortality (95% CI: 6.1, 9.5%), compared with a 3.3% increase (95% CI: –1.3, 8.2%) in the present study. In our study, unadjusted associations between relative-apparent-temperature–defined heat waves (HI08, HI09, and HI10, with maximum daily apparent temperature thresholds of > 85th, > 90th, and > 95th percentile, respectively) followed patterns similar to those reported by Hattis et al. (2012) in Massachusetts: More extreme heat waves had larger associations (Figure 1), although the associations found in the present study (1.2–2.7%) were smaller than those reported by Hattis et al. (2012) (3.7 to 5.3%).

*Examination of overall heat wave effects among HIs*. Our results suggest that significant positive associations were more commonly present when relative, compared with absolute, HIs were used to define exposure. This suggests that public health warning systems may be more effective using regionally specific definitions. This finding is consistent with other studies (Knowlton et al. 2007) and suggests that reliance on the absolute measures of the National Weather Service alert system (HI13–HI16) may not be optimal for protecting public health.

Previous physiological research shows that high temperatures, as well as increased humidity, heighten the risk of heat illness (Coris et al. 2004; Perry et al. 2011). However, findings in the present study suggest that HIs including humidity and other meteorological factors [i.e., heat waves defined using apparent temperature (HI08–HI10) or heat index values HI13–HI16)] may not be more predictive of adverse heat-related health effects, which is consistent with previous research (Vaneckova et al. 2011). In addition, minimum or nighttime temperature has been shown to be predictive of mortality (Anderson and Bell 2011; D’Ippoliti et al. 2010), possibly because of decreased recovery time or association with humidity. We evaluated one metric based on minimum temperature alone (HI05; minimum daily temperature > 95th percentile for ≥ 2 consecutive days), which had a nonsignificant positive association with PTB. Mean temperatures, which reflect both minimum and maximum temperatures, seem to be more predictive of health outcomes in the present study, which is consistent with previous studies (Anderson and Bell 2009; Barnett et al. 2010; Vaneckova et al. 2011).

Model power is variable because of the different number of classified heat wave days between HIs, due not only to the observed effect–measure strength but also to the total number and geographical distribution of heat wave days. However, in comparing point estimates of associations between heat waves and health outcomes, there are patterns in results of mortality models using HIs with similar proportions of cases on heat wave days across the 20-year period of study (HI02, HI06, HI10, HI11, and HI15; Table 1). From NAD models containing these five HIs, those with relative-scale cut-point definitions (HI02, HI06, and HI10) showed similar positive associations and had lower confidence limits that were above or near zero (Figure 1B). Models with absolute-scale cut-point definitions (HI11 and HI15) resulted in negative associations closer to zero. In PTB models using these five heat wave definitions, relative-scale cut-point HI-defined heat wave days had positive (HI02 and HI06) or near-null (HI10) associations, and absolute-scale cut-point HI-defined heat wave days had negative associations (Figure 1A).

Previous and current research highlights the importance of the choice of HI for interpretation of health effects related to climate change. Based on the current and previous analyses (Anderson and Bell 2009; Vaneckova et al. 2011), HIs based on mean daily temperature (such as HI01–HI03) may be the most useful and simplest metric for the United States, although more comparative analyses at the regional level are needed. Smith et al. (2012) suggested that the Southeast, compared with the rest of the United States, has experienced the most widespread increase in heat wave days from 1979 to 2011 across the majority of HIs (Smith et al. 2013). Specifically, increases in HI01, HI02, HI06, HI07, HI08, HI09, HI11, HI14, and HI15 have been greater in the Southeast than in most other U.S. regions. HI02, HI06, HI09, HI11, and HI14 showed more than a half-day average increase in the yearly number of heat wave days within the Southeastern United States over the 1979 to 2011 period. From the perspective of projecting future climate change impacts, choosing HIs based on relative daily temperatures from these five metrics (i.e., HI02 and HI06) has a practical advantage because they show positive associations with adverse health outcomes, and because projecting daily air temperature is a simpler (although not trivial) problem relative to projecting multiple meteorological variables at subdaily time scales, as is required by the more complex HIs.

*Comparison of heat wave characteristics across HIs*. Previous studies have suggested that associations between heat wave days and mortality are stronger during heat waves of longer duration and during the first heat wave of the season (Anderson and Bell 2011). Results of the present study suggest that longer heat waves are associated with increased magnitude of the positive associations with NAD, although seasonality patterns were not found (Figure 3; see also Supplemental Material, Figure S2; data not shown for seasonality).

*Effect–measure modification by rurality across HIs*. Recent literature focused on urban areas suggests that urban regions have higher mortality risks from heat waves, although rural areas are rarely studied (Basu 2009). Based on numerous studies, prominent risk factors for heat wave–associated mortality include social isolation, poverty, and age > 65 years (Ebi et al. 2006; English et al. 2009; Haines et al. 2006), all of which are prevalent in many rural areas. Yet previous studies have used exposure variables (e.g., land surface temperature) in urban–suburban areas, not rural areas (Hondula et al. 2012; Johnson et al. 2009). Heat wave warning systems that use relative thresholds are typically based on average temperatures in relatively large regions containing both urban and rural areas (Lowe et al. 2011). Results of the present study do not suggest strong effect modification by rurality for heat wave–related PTB or NAD. Although NAD risk may be heightened in urban areas, with positive associations consistent in urban areas but not in more rural areas, most interaction terms between heat waves and rurality were not significant (*p* > 0.05). All significant rurality-specific associations were positive.

*Potential limitations*. As with previous studies examining relationships between heat waves and health outcomes, we did not have a measure of time spent outdoors to determine the degree to which subjects were exposed to ambient temperatures. Although air conditioning has been found to modify the association between temperature and mortality (Anderson and Bell 2009), as with most heat wave studies, we did not have a measure of air conditioning. Also, we did not adjust for air pollution. However, Anderson and Bell (2009) found that relationships between temperature and mortality remained after adjustment for air pollution exposure. The present study includes data only from Alabama, so it is not generalizable to other regions with different climates, demographics, and housing characteristics. As in most previous heat wave studies, we did not adjust for the multiple comparisons that were examined.

## Conclusions

Our results suggest that previous findings of associations between heat waves and adverse health outcomes may also apply in the state of Alabama, in the southeastern United States. However, associations were highly variable depending on the measure used to define heat waves. Our findings show that the use of different HIs can result in different association estimates when studying health effects of extreme heat events. These findings have implications for operational warning systems and for studies designed to quantify health impacts of climate change. Interestingly, it was not clear whether including multiple health-relevant meteorological parameters in an index improved model fits or whether use of these parameters was more likely to find significant associations, suggesting that simple, temperature-only indices might be most appropriate for use in warning systems and for climate impacts analysis. Finally, heat wave days were associated with PTB and NAD in both rural and urban areas, depending on the heat wave definition used, highlighting the need to develop heat wave response systems in both cities and rural areas.

## Supplemental Material

(324 KB) PDF
