# Supplementary material for: Heat Waves and Health Outcomes in Alabama (USA): The Importance of Heat Wave Definition
Source: Environ Health Perspect. 2013 Nov 22;122(2):151–8. doi: 10.1289/ehp.1307262 (PMC3914868; doi:10.1289/ehp.1307262)
Supplement: (324 KB) PDF [file ehp.1307262.s001.508.pdf]

Environmental Health Perspectives doi:10.1289/ehp.1307262

Supplemental Material

Heat Waves and Health Outcomes in the Alabama (USA): The Importance of Heat Wave Definition

Shia T. Kent, Leslie A. McClure, Benjamin F. Zaitchik, Tiffany T. Smith, and Julia M. Gohlke

Table of Contents

Supplemental Material, Table S1……………………………………………….…Page 2

Supplemental Material, Table S2………………………………………………….Page 3

Supplemental Material, Figure S1……………………………………..……..Page 4

Supplemental Material, Figure S2…………………………………………..…..Page 5

Supplemental Material, Table S1. Percent changes in preterm birth or non-accidental death risk on a heat wave day compared to non-heat wave days, unadjusted and adjusted for daily mean temperature.

	**Preterm birth**		**Non-accidental death**	
**HI**	**Unadjusted**	**Temperature-adjusted**	**Unadjusted**	**Temperature-adjusted**
HI01	11.6% (0.9, 23.4)	6.9 (-4.4, 19.5)	1.5% (-3.5, 6.7)	-2.2% (-7.4, 3.3)
HI02	1.5% (-3.7, 6.9)	-4.9% (-11.2, 1.9)	3.7% (1.1, 6.3)	1.5% (-1.7, 4.8)
HI03	32.4% (3.7, 69.1)	26.1% (-1.6, 61.7)	-3.3% (-14.0, 8.7)	-6.8% (-17.2, 5.0)
HI04	-33.3% (-92, 453.9)	-36.5% (-92.4, 426.7)	-17.8% (-57.8, 59.9)	-19.6% (-58.6, 56.2)
HI05	34.6% (-4.8, 90.4)	27.1% (-10.4, 80.3)	-5% (-23.6, 18.0)	-8.5% (-26.4, 13.8)
HI06	3.8% (-3.0, 11)	0.2% (-7.5, 7.6)	1.9% (-1.4, 5.2)	-0.6% (-4.1, 2.9)
HI07	9.5% (-0.6, 20.7)	5.8% (-4.6, 17.3)	5.5% (1.0, 10.2)	3.3% (-1.3, 8.2)
HI08	0.5% (-2.3, 3.5)	-1.5% (-4.7, 1.8)	1.2% (-0.1, 2.6)	0.1% (-1.4, 1.7)
HI09	-1.5% (-5.0, 2.1)	-4.7% (-8.5, -0.6)	2% (0.3, 3.8)	0.8% (-1.2, 2.7)
HI10	0.3% (-5.6, 6.4)	-3.7% (-9.8, 2.9)	2.7% (-0.2, 5.7)	0.7% (-3.8, 2.5)
HI11	-8.9% (-17.6, 0.8)	-9.2% (-17.9, 0.4)	-0.7% (-5.4, 4.1)	-0.8% (-5.4, 4.1)
HI12	-9.7% (-16.4, -2.5)	-9.8% (-16.4, -2.5)	-1.5% (-5.0, 2.2)	-1.4% (-4.9, 2.3)
HI13	1.3% (-1.5, 4.2)	1.2% (-1.6, 4.1)	-0.4% (-1.6, 0.8)	-0.5% (-1.7, 0.8)
HI14	-0.4% (-2.6, 1.8)	-0.5% (-2.7, 1.7)	-0.1% (-1.1, 0.9)	-0.2% (-1.2, 0.8)
HI15	-4% (-9.8, 2.3)	-4.1% (-10.0, 2.1)	-1.3% (-4.4, 1.8)	-1.2% (-4.3, 1.9)

Estimates are derived from odds ratios and 95% CI estimated using case-crossover conditional logistic regression models. Temperature was adjusted for by modeling daily mean temperature as a natural cubic spline with three degrees of freedom and equally spaced knots. HI definitions can be found in the main text, Table 1.

Supplemental Material, Table S2. Differences in Akaike Information Criterion (AIC) values for models examining preterm birth (PTB) and non-accidental death (NAD) risk on a heat wave day, by 15 heat wave indices (HIs).

HI	PTB	NAD
HI01	2.35	7.46
HI02	6.57	0
HI03	1.92	7.46
HI04	6.72	7.44
HI05	4.18	7.57
HI06	5.71	6.52
HI07	3.51	2.03
HI08	6.74	4.61
HI09	6.21	2.55
HI10	6.87	4.57
HI11	3.55	7.69
HI12	0	7.14
HI13	6.09	7.32
HI14	6.74	7.72
HI15	5.28	7.1

Differences in AIC values are between each HI and the lowest HI for either PTB (HI12) or NAD (HI02). AIC values are derived unadjusted case-crossover conditional logistic regression models comparing PTB or NAD on heat wave days versus corresponding non-heat wave control days.

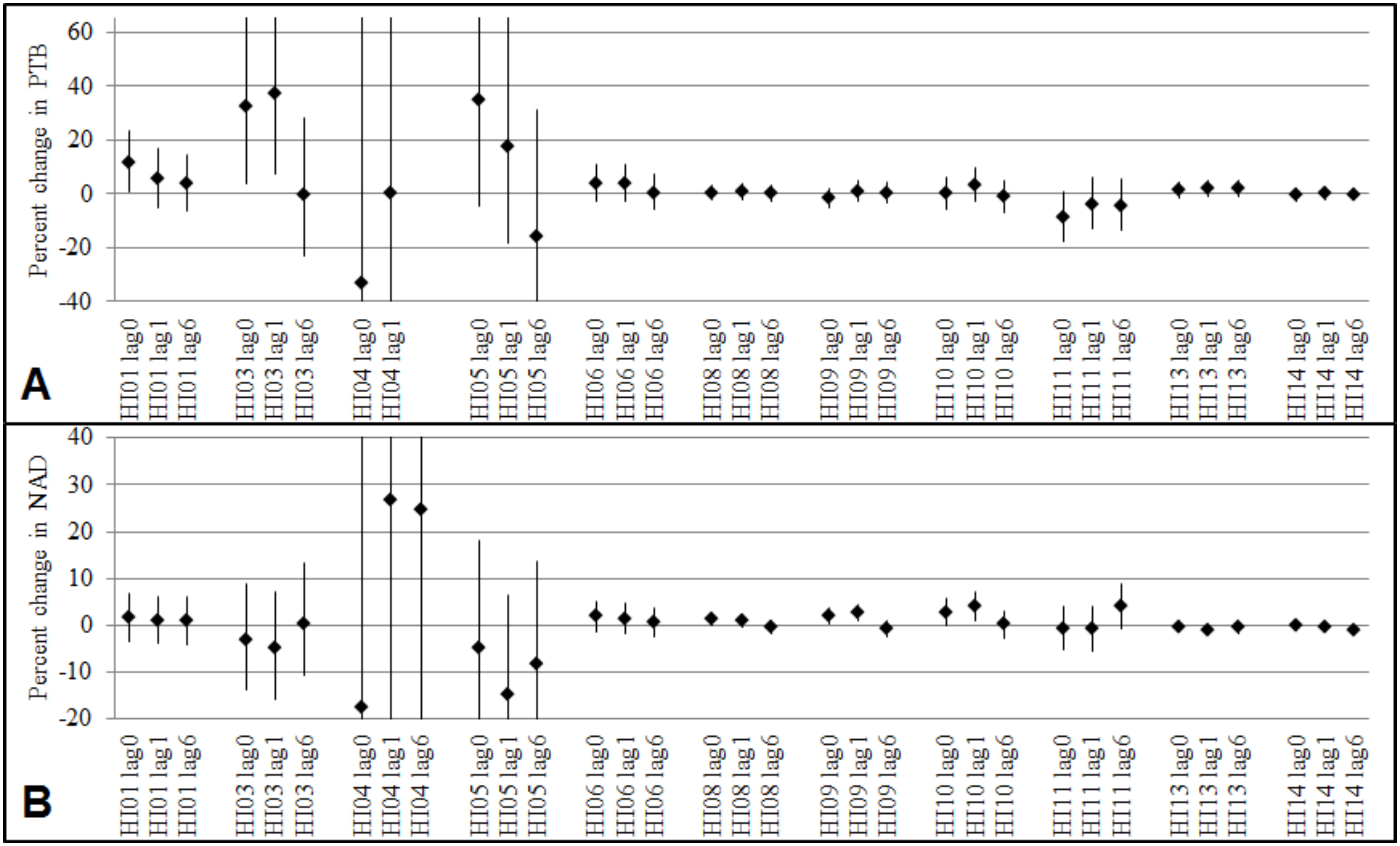

Supplemental Material, Figure S1. Percent changes in (A) preterm birth [PTB] or (B) non-accidental death [NAD] for lag0, lag1, and lag6 models, for heat indices not displayed in Figure 1. Estimates are derived from odds ratios and 95% CI estimated using case-crossover conditional logistic regression models comparing PTB or NAD on heat wave days versus corresponding non-heat wave control days. PTB model HI04 lag6 estimates could not be calculated due to insufficient model power.

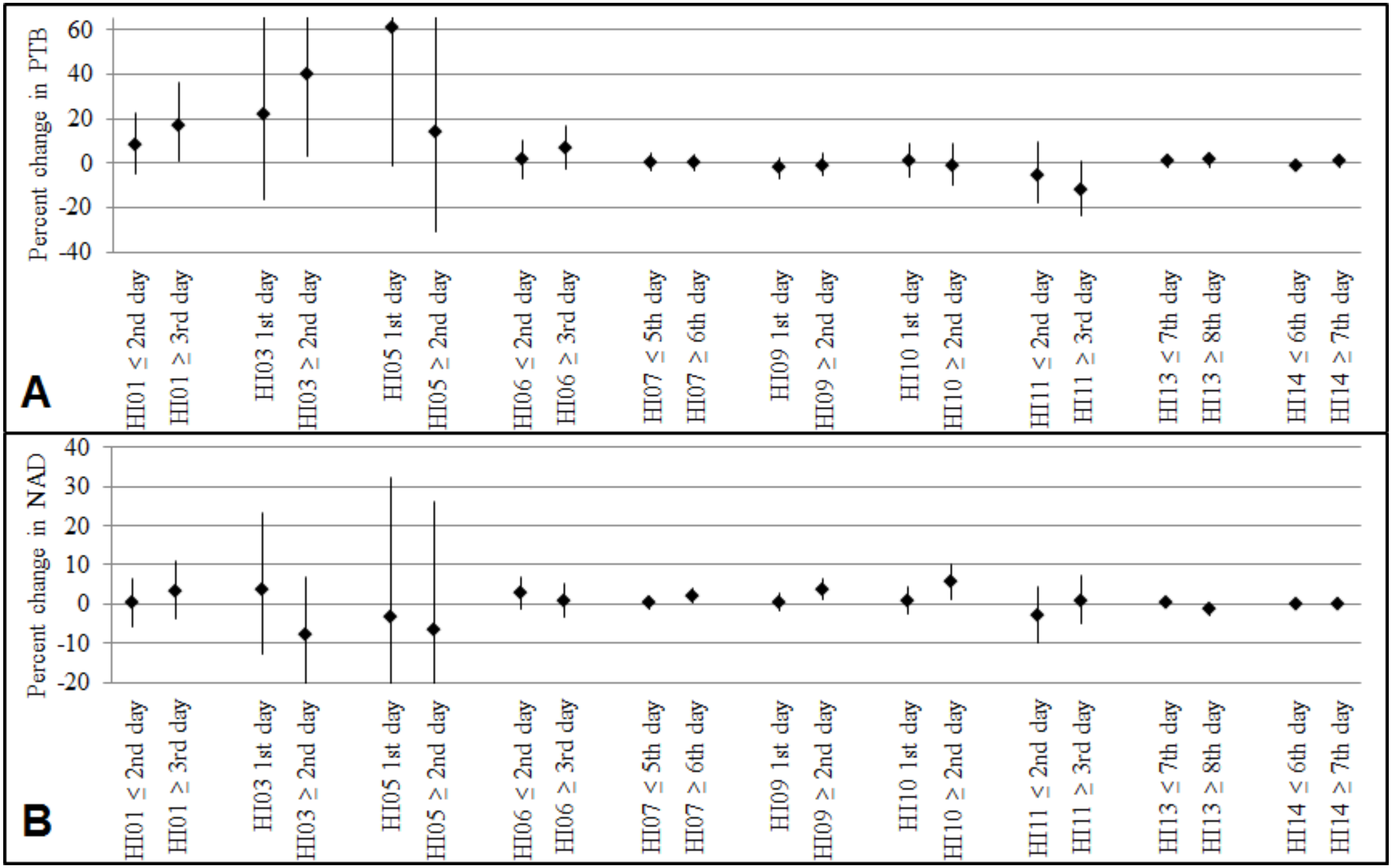

Supplemental Material, Figure S2. Percent changes in (A) preterm birth [PTB] or (B) non-accidental death [NAD] for heat wave duration models, for heat indices not displayed in Figure 1. Estimates are derived from odds ratios and 95% CI estimated using case-crossover conditional logistic regression models comparing PTB or NAD on heat wave days versus corresponding non-heat wave control days. HI04 estimates could not be calculated due to insufficient model power.
